# Prescience of endogenous regulation in *Arabidopsis thaliana* by *Pseudomonas putida* MTCC 5279 under phosphate starved salinity stress condition

**DOI:** 10.1038/s41598-020-62725-1

**Published:** 2020-04-03

**Authors:** Sonal Srivastava, Suchi Srivastava

**Affiliations:** 1 Division of Microbial Technology, CSIR-National Botanical Research Institute, Rana Pratap Marg, Lucknow, 226 001 India; 2 Academy of Scientific and Innovative Research, AcSIR, Ghaziabad, 201002 India

**Keywords:** Microbiology, Abiotic

## Abstract

Phosphorus (P) availability and salinity stress are two major constraints for agriculture productivity. A combination of salinity and P starvation is known to be more deleterious to plant health. Plant growth promoting rhizobacteria are known to ameliorate abiotic stress in plants by increasing the availability of different nutrients. However, interaction mechanisms of plant grown under salinity and P stress condition and effect of beneficial microbe for stress alleviation is still obscure. Earlier we reported the molecular insight of auxin producing, phosphate solubilising *Pseudomonas putida* MTCC 5279 (RAR) mediated plant growth promotion in *Arabidopsis thaliana*. In present study new trait of proline and phosphatase production of RAR and its impact on modulation of physiological phenomenon under phosphate starved-salinity stress condition in *A. thaliana* has been investigated. Different physiological and molecular determinants under RAR- *A. thaliana* interaction showed that auxin producing RAR shows tryptophan dependence for growth and proline production in ATP dependant manner under salinity stress. However, under P deprived conditions growth and proline production are independent of tryptophan. RAR mediated lateral root branching and root hair density through modulation of abscisic acid signalling was observed. Acidic phosphatase activity under P starved and salinity stress condition was majorly modulated along with ROS metabolism and expression of stress responsive/phosphate transporter genes. A strong correlation of different morpho-physiological factor with RAR + salt conditions, showed We concluded that enhanced adverse effect of salinity with unavailability of P was dampened in presence of *P. putida* MTCC 5279 (RAR) in *A. thaliana*, though more efficiently salinity stress conditions. Therefore, alleviation of combined stress of salinity induced phosphate nutrient deficiency by inoculation of beneficial microbe, *P. putida* MTCC 5279 offer good opportunities for enhancing the agricultural productivity.

## Introduction

Soil salinity, an important global problem occupies more than 7% of the earth’s land surface. Approximately 7 million hectare of land in India is covered by saline soil with an yearly increase @ 0.3–1.5 million ha of farmland^1,2^. It has been reported as most serious factor for limiting the productivity of agricultural crops by 20%^3,4^. Numerous hostile effects such as osmotic stress, ion toxicity, hormonal imbalance and generation of reactive oxygen species are known to be induced by salinity stress in plants^5^. These alterations are known to affect the growth and development of plant by impairing the nutritional balance. Among different nutrients, salinity stress are known to limit the phosphorus (P) availability, uptake and transport in plants probably due to its precipitation with other cations and hindrance of the acidic phosphatase activity^6–8^.

Phosphorus (P) is one of the major essential macronutrient, directly taken up by the plants in form of HPO_4_^−^ ions. However, majority of it gets immobilized in forms of Fe, Al and Ca phosphates in soil. The unavailable form of immobilized soil P gets hydrolysed to inorganic P by the production of organic acid and phosphatase enzymes^9,10^. Phosphatases are believed to be important for phosphorous scavenging and remobilization in plants by the process of P mineralization^11^. These enzymes are widely found in plants and catalyze reactions in both acidic and alkaline medium^12^.

Likewise plants, microbes are well known for producing phosphatase enzymes and constitute the largest proportion of extracellular soil phosphatases^13,14^. These microbes are integral part of soil phosphorus recycling and its availability to the plants^15^. Majority of rhizospheric and soil microorganisms mineralize P by the action of phosphatases. Number of microbes belonging to different genera (*Bacillus*, *Streptomyces*, *Pseudomonas* etc.) are known for mineralization of organic phosphates^16^. They solubilize precipitated phosphates and serve as a sink for P by rapidly immobilizing even in P starved conditions. However, the role of these phosphatases in adaptation to abiotic stresses has not been critically evaluated.

Plant growth-promoting bacteria of different traits such as phosphate solubilisation, auxin and proline production are known to improve plant growth *via* diverse mechanisms. They are known to sustain biomass, photosynthesis and homeostasis of toxic ions under saline stress^17,18^. Change in soil P dynamics and better availability of phosphate to plants is a general beneficial effect of phosphate solubilising bacteria^19^. On the other hand auxin producing microbes modify the root architecture of plant to access more nutrients and water from the soil^20,21^. However, auxin production depends on presence of its precursor, tryptophan, to regulate various physiological processes of the plants^21^. Proline catabolism has also been reported as determinant for rhizosphere colonization in *P. putida* by Vilchez *et al*.^22^. Enhanced uptake of P through AMF colonization by the involvement of different mechanisms such as secretion of acid and alkaline phosphatases, maintenance of polyphosphate concentration and expression of high affinity phosphate transporter genes has been reported earlier^23^.

Management of mineral nutrients to alleviate salt induced nutritional disorders in plants is one of the main to improve crop salt tolerance and productivity^24^. Less P availability, rising costs of P fertilizer, lesser P use efficiency of the plant^25^ justifies the need to explore the microbial potential to increase the availability of P for developing a more sustainable agriculture strategies. Renewed emphasis on providing sufficient food for a growing world population, different aspects of rhizobacteria-plant interaction are gaining importance^26^. Among different attempts, an exogenous application of phosphate solubilising and phosphatase producing microbes have been reported to promote plant growth^27,28^. Potentiality of P-biofertilizer *Azolla* under salinity stress condition by the involvement of acid phosphatase activity has also been reported earlier^8^. Therefore, utilization of microorganisms with above attributes is an attractive proposition to improve the plant growth under stress condition by maintaining nutritional balance. Interactions between microorganisms and plants in soil environments are complex and the role of *Pseudomonas putida* MTCC 5279 (RAR) for plant growth promotion through modulation of different genes has already been reported^29^. Further, in present study the role of RAR as phosphatase and proline producer for plant growth promotion under phosphate starvation combined with salinity stress condition has been studied.

## Results

Role of P solubilising, auxin producing *P. putida* MTCC5279 has been reported earlier for its plant growth promoting attribute using *A. thaliana* as a host plant^29^. The objective of present study is to evaluate the interconnection between different traits of RAR and host plant (*A. thaliana)* under combined stress of salinity and phosphate. Results are summarized under following heads-

### Effect of salinity and phosphate sources on growth of *P. putida* MTCC 5279

Plant-growth-promoting rhizobacteria RAR, didn’t show any alteration in growth pattern while grown in M9 minimal medium supplemented upto 500 mM NaCl (Supplementary Figs. S1, S2A). Higher concentration of salt (1 M NaCl) was found to negatively affect the growth of the strain by extending its lag phase by a period of 7 days (Supplementary Fig. S2A). On the other hand limited P conditions (0.3% KH_2_PO_4_) was found to enhance the stress severity of salt (1 M NaCl + 0.3% KH_2_PO_4_) and RAR attained the early death at 7^th^ day as compared to alone NaCl (1 M) conditions (Supplementary Fig. S2B). Interestingly, presence of insoluble source of phosphate (Tri Calcium Phosphate; TCP), RAR with extended lag phase survived up to 7 days in presence of salt (1 M NaCl + 1.5% TCP) (Supplementary Fig. S2C). Presence of tryptophan (TPP; a precursor for auxin biosynthesis) minimized the effect of salt stress, however, its absence was found to negatively affect the growth of RAR (Supplementary Fig. S2A–C).

### Proline accumulation in *P. putida* MTCC 5279 under salinity and phosphate stress conditions and effect of auxin precursor (tryptophan) and ATP inhibitor

Proline accumulation has been found as a common phenomenon of RAR. Higher accumulation of proline at 24 h of incubation was found to be reduced on prolonged incubation of 7 day under all P conditions (1.2% Na_2_HPO_4_ + 0.3% KH_2_PO_4_, 0.3% KH_2_PO_4_ and 1.5% TCP) (Supplementary Fig. S2A–C). Role of proline as stress marker was evident after 5^th^ day of incubation and enhanced proline accumulation can be seen under stressed conditions as compared to control. Less proline accumulation at 500 mM NaCl got induced after 5^th^ day as compared to RAR grown in M9 medium. Similar pattern of proline accumulation in RAR with 1 M salt concentration at normal and unavailable (TCP) P conditions was observed (Supplementary Figs. S2A,C). Among the three P conditions, proline accumulation was highest in limited P (0.3% KH_2_PO_4_) (Supplementary Fig. S2B). RAR produced lower amount of proline in absence of tryptophan (TPP) under all growth conditions and differences was more prominent in presence of insoluble source of P (TCP), thereby showing the dependence of proline production with TPP.

Determination of the role of ATP inhibitor carbonyl cyanide-m-chlorophenylhydrazone (CCCP) in proline and P-accumulation of RAR showed inverse relation of proline with accumulated Pi under normal and salinity stress condition (Supplementary Fig. S3A). Both quantitative and qualitative methods showed reduced proline production in presence of CCCP showing the role of ATP in proline production (Supplementary Fig. S3B,C). Gain of intense pink colour (proline production) under salinity stress condition was observed in absence of CCCP, while, no intense pink colour was observed under salinity stress condition in presence of CCCP probably due to metabolic shift (Supplementary Fig. S3C).

### Hormone production in *P. putida* MTCC 5279 under salinity and phosphate stress conditions

Hormone production was determined in the liquid culture of RAR grown under P starved-salinity stress condition in presence and absence of auxin precursor, tryptophan. Under normal conditions of growth, RAR produced auxin both in absence and presence of its precursor. Both stresses *viz*. salinity (500 mM and 1 M salt) and limited phosphate (0.3% KH_2_PO_4_) showed negative correlation with IAA production as compared to control. In presence of insoluble P (TCP; 0.71 µg/g biomass), auxin production was similar to the control (0.72 µg/g biomass). However, auxin production under stress condition was greatly affected by the absence of tryptophan. Pattern of gibberellic acid was almost similar to the auxin indicative of some common link between gibberellins and auxin production (Supplementary Fig. S4A,B).

### Correlation among different attributes of *P. putida* MTCC5279 under salinity and phosphate stress conditions

**Figure 1:**
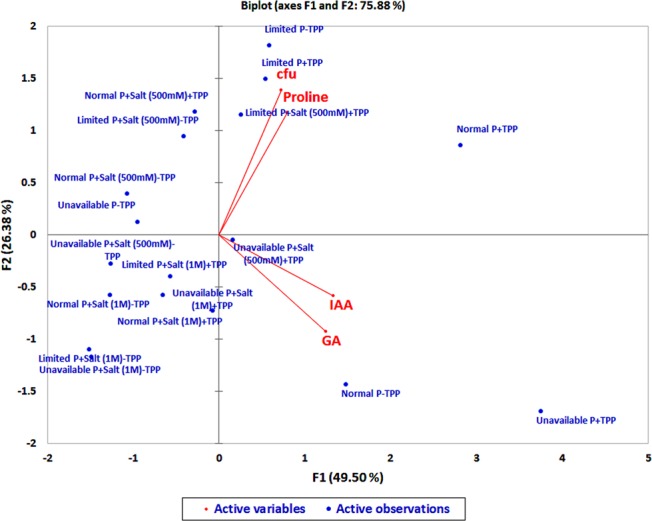
Biplot graph to draw correlation among different traits of *Pseudomonas putida* MTCC 5279 (RAR) grown under conditions of salinity (0, 500 mM and 1 M NaCl) and phosphate sources *viz*. Normal P (0.3% KH_2_PO_4_ + 1.2%Na_2_HPO_4_), limited P (0.3% KH_2_PO_4_) and unavailable P (Tri calcium phosphate) in presence of auxin precursor tryptophan (TPP).

Relatedness of different plant growth promotary attributes *viz*. growth, proline, IAA and GA production of RAR grown under different conditions of salinity and P starvation after 48 h of incubation were determined through principal components analysis (PCA) (Fig. 1). The percent variability was found to be higher due to F1 factor (49.50%) as compared to F2 factor (26.38%). Biplot categorized the traits into two groups *i.e*. group 1 consisted of cfu/ml and proline and group 2 with IAA and GA. Within the group, traits had strong positive correlation with each other, however, no such correlation was observed among groups (Fig. 1). Different treatments were found to be distributed in different coordinates. Treatments present away from the origin in positive direction *i.e*. normal P + TPP, limited P +/−TPP, limited P + salt (500 mM)+TPP, unavailable P + salt (500 mM) + TPP, normal P-TPP and unavailable P + TPP showed their better correlation with above mentioned traits of RAR as compared to the treatments farther away from origin in negative direction. Result shows the correlation of TPP with proline production under normal and limited P condition. A clear relationship between proline production and growth (CFU/ml) of RAR showed TPP independence under P stress conditions. However, TPP was found to help the growth under lower salt stress (500 mM) conditions. IAA and GA production are found correlated with normal P conditions under TPP deprived condition (Fig. 1).

### Phosphatase activity of *P. putida* MTCC5279 under salinity and phosphate stress conditions

**Figure 2:**
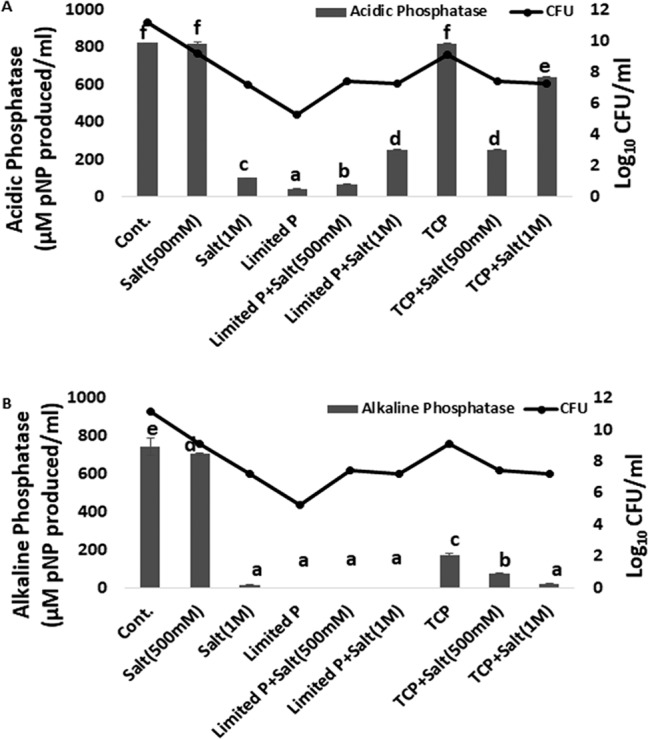
Effect of salinity (0, 500 mM and 1 M, NaCl) and different phosphate sources [(Normal P (0.3% KH_2_PO_4_ + 1.2%Na_2_HPO_4_), limited P (0.3% KH_2_PO_4_) and unavailable P (Tri calcium phosphate)] stress on phosphatase activity (**A**) acidic phosphatase (**B**) alkaline phosphatase. Results are the means of three independent experiments. Vertical bars indicate mean ± S.D. of three replicates.

Production of phosphatase as a measure of alkaline phosphate solubilizer was measured in *P. putida* MTCC5279 (RAR) under salinity stress conditions. Phosphatase activity of RAR was not found to be affected by 500 mM NaCl concentration, whereas, 1 M NaCl negatively affected both alkaline and acidic phosphatase activity. P limitation (0.3% KH_2_PO_4_) negatively affected the acidic and alkaline phosphatase activity in absence and presence of salt. Insoluble P source (TCP), reduced the alkaline phosphatase activity under 0, 500 and 1 M salt conditions, however, acidic phosphatase activity in presence of TCP was similar to normal P. Higher concentration of salt (1 M) was found to enhance the acidic phosphatase activity in presence of both limited and insoluble P conditions (Fig. 2A,B).

### Effect of *P. putida* MTCC5279 inoculation on growth of *A. thaliana* under phosphate starved-salinity stress condition

**Figure 3:**
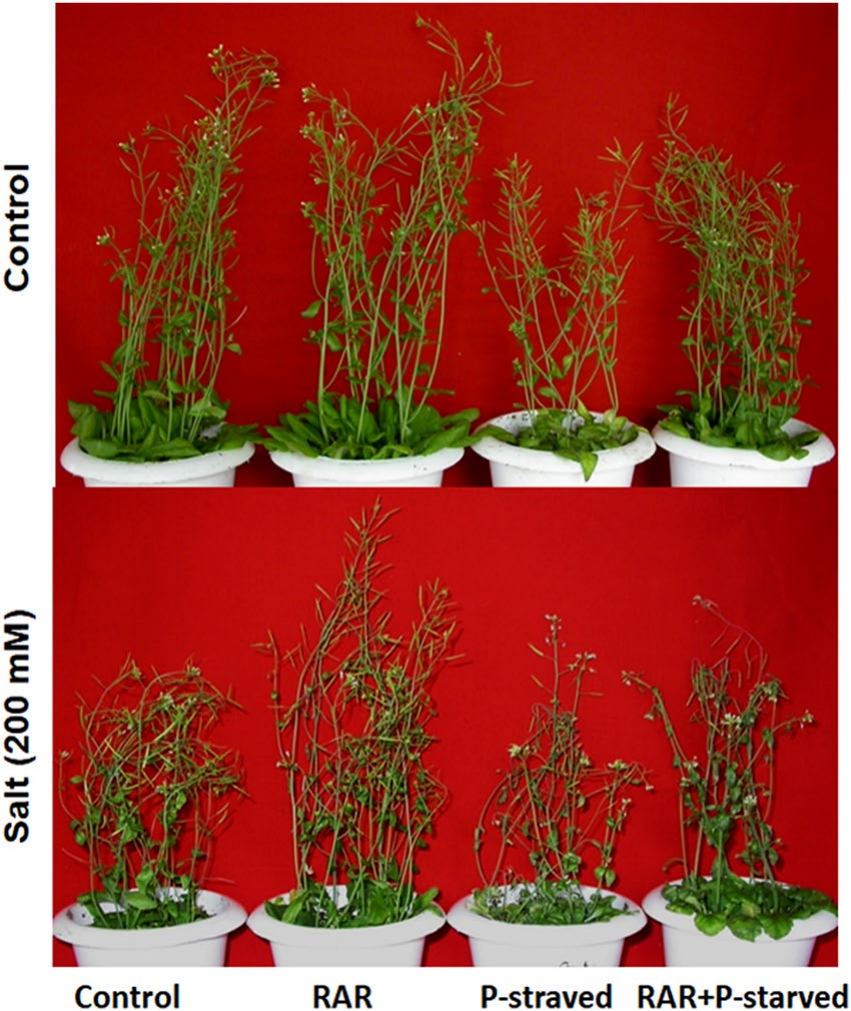
Effect of phosphate starvation and salinity stress on *Arabidopsis thaliana* in presence of *Pseudomonas putida* MTCC 5279 (RAR).

Effect of salinity stress (50–500 mM) and P starvation resulted in reduced germination, growth and altered accumulation of hormones in *A. thaliana* (Col-0) (Supplementary Fig. S5A,B). The effect of RAR inoculation on *A. thaliana* (Col-0) under such stress conditions resulted in increase in vegetative growth of plants (Fig. 3; Supplementary Fig. S6). Significant increase in plant height (25.88%), number of leaf (72.34%) and dry weight (104%) was noted in RAR inoculated *A. thaliana* as compared to uninoculated plants. Inoculation of RAR also enhanced the formation of siliques by 21.17% as compared to control plants (Fig. 3; Supplementary Table 2). However, stresses like phosphate, salinity and P starved-Salinity negatively affected the dry weight of *Arabidopsis* plant by 39.53, 44.18 and 57.77%, respectively. Significant increase in dry weight by 65.11, 30.76 and 36.84% under phosphate, salinity and salinity-phosphate stressed conditions by the presence of RAR as compared to their respective controls was observed. Number of siliques monitored as indicator of productivity got reduced by 49.11%, 35.88% and 88.80% due to the imposed stressed conditions of phosphate, salinity and P starved-salinity, respectively. Higher number of silique formation by 52.02, 48.57 and 263.00% in presence of RAR as compared to their respective controls, with better seed yield, was observed (Supplementary Table 2).

Effect of *P. putida* inoculation on proline accumulation in *A. thaliana* leaves in relation to P availability showed that under normal phosphate conditions, RAR enhanced the proline (111.95 µM) accumulation in order to ameliorate the imposed salt stress as compared to control (29.70 µM). However, lower proline accumulation was found under phosphate starved-salinity stress (133.51 µM) conditions as compared to salt stressed conditions (209.68 µM). Under phosphate starved conditions, proline accumulation was ~5 fold higher as compared to normal phosphate conditions (Supplementary Table 2). Reduction of proline accumulation under P-starved condition in presence of RAR was found to be enhanced under salinity and combined stress of both salinity and P. Probably lesser accumulation of proline under P stress condition as compared to P starvation is contributing to RAR mediated alleviation of P stress by increasing p availability (Supplementary Table 2). Phosphate starvation resulted in 54.42% lower accumulation of phosphate as compared to control. Similarly, salinity stressed plants showed lower phosphate content (143.00 µg/g FW) as compared to control (181.48 µg/g FW), however, RAR inoculation resulted in 24.92 and 1.14% increase in phosphate uptake under salt and P starved-salinity stress condition, respectively (Supplementary Table 2).

**Figure 4:**
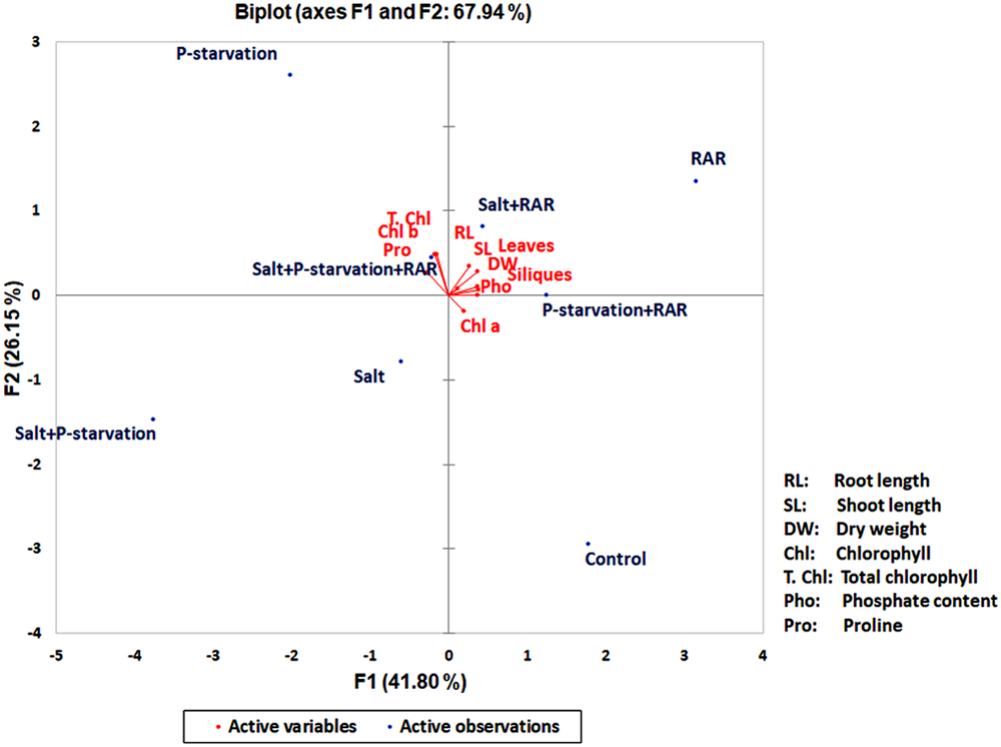
Correlations among different physiological parameters of the plants grown under salinity, phosphate starved and phosphate starved-salinity stress conditions through biplot PCA analysis.

The correlations among different physiological parameters of the plants grown under different conditions were studied through biplot analysis (Fig. 4). Disparity among different growth conditions and their relatedness with physiological parameters of plants in presence and absence of RAR was clearly evident through biplot. The PCA comprising two principal components (F1: 41.80% and F2: 26.15%) accounted for 67.94% of variance. Treatments such as salt, p-starvation and salt + p-starvation were distributed in left quadrants *i.e*. in the negative direction. However, treatments inoculated with RAR *i.e*. RAR, salt + RAR, P-starvation + RAR were present away from the origin in positive direction. Plant physiological parameters *viz*. root length, shoot length, phosphate content, number of siliques, leaves, proline and chlorophyll content were found to be closely associated with treatments inoculated with RAR. Distribution of active variables and observations shows direct involvement of RAR in improving plant growth through enhancement of nutrient acquisition, growth and productivity of plants under salinity and P starved-salinity stress condition (Fig. 4). However, the plant growth promotary response of RAR was more efficient under salt stress in comparison to P deficient conditions.

### Effect of *P. putida* MTCC5279 inoculation on root architecture of *A. thaliana*

**Figure 5:**
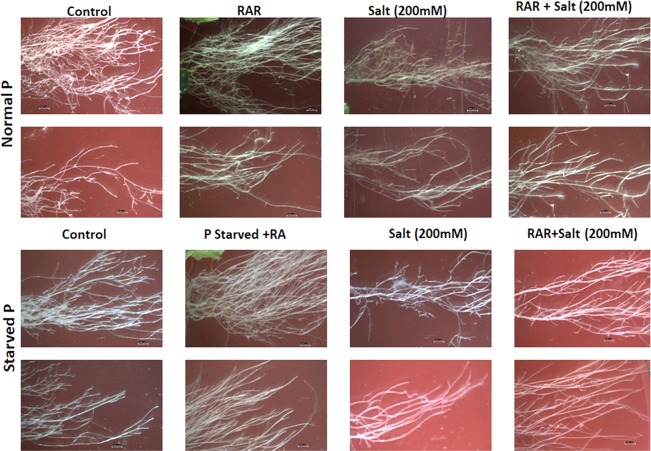
Effect of *Pseudomonas putida* MTCC 5279 (RAR) inoculation on the root architecture of *A. thaliana* grown under salinity, phosphate starved and phosphate starved-salinity stressed (NaCl, 200 mM) condition.

*P. putida* (RAR) treatment was found to enhance the emergence of number of roots of *A. thaliana* grown  in petriplate conditions (Supplementary Fig. S6A). Under hydroponic conditions similar result of better root proliferation was observed under non-stressed conditions (Fig. 5; Supplementary Fig. S6). RAR mediated better root architecture both in terms of enhanced number of lateral roots as well as better growth of main root was observed. Lateral root growth was more hampered under salinity stress condition as compared to the P stress alone. However, when both stress were applied together their combined effect was more adverse as compared to the alone stress conditions. PGPR inoculation under these conditions improved the root density (Fig. 5).

### Effect of *P. putida* inoculation on modulation of ROS species in *A. thaliana*

**Figure 6:**
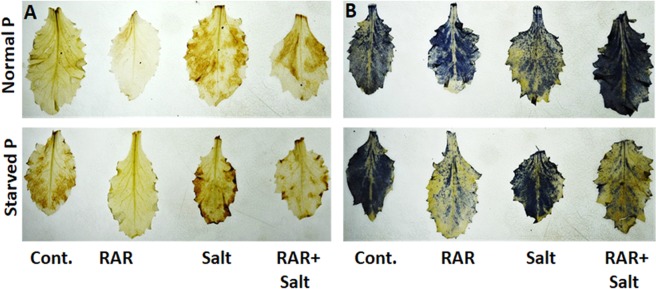
Modulation of ROS species (O_2_^−^, H_2_O_2_) in *A. thaliana* leaves by the inoculation of *Pseudomonas putida* MTCC 5279 (RAR) under salinity, phosphate starved and phosphate starved-salinity stressed (NaCl, 200 mM) condition through DAB and NBT staining.

Under standard condition (Normal P control), a week DAB staining in the leaves of *A. thaliana* was observed as compared to P starved condition (Fig. 6). Nonetheless, exposure to salinity stress (200 mM) resulted in intense staining, indicating high accumulation of H_2_O_2_ under salinity stress condition. Presence of RAR under salinity stress reduced the accumulation of H_2_O_2_ in leaves. The NBT staining results revealed that plants grown under P starvation condition accumulated more superoxide radical as compared to control (Fig. 6). Combined stress of P starvation and salinity showed intense NBT staining as compared to plants grown under alone salinity stress condition. However, inoculation of RAR lowered its accumulation under both P starved as well as P starved-salinity stressed condition. Activities of catalase and APX was measured in shoot tissue grown under P starved-salinity condition. More pronounced alteration in catalase activity under stress condition both in presence and absence of RAR was observed, however, RAR alone didn’t show altered activity. Maximum activity was observed under salt + RAR condition. APX activity gets induced under P starvation both in presence and absence of RAR (Supplementary Fig. S7).

### Effect of *P. putida* inoculation on IAA, GA3 and ABA accumulation in *A. thaliana*

**Figure 7:**
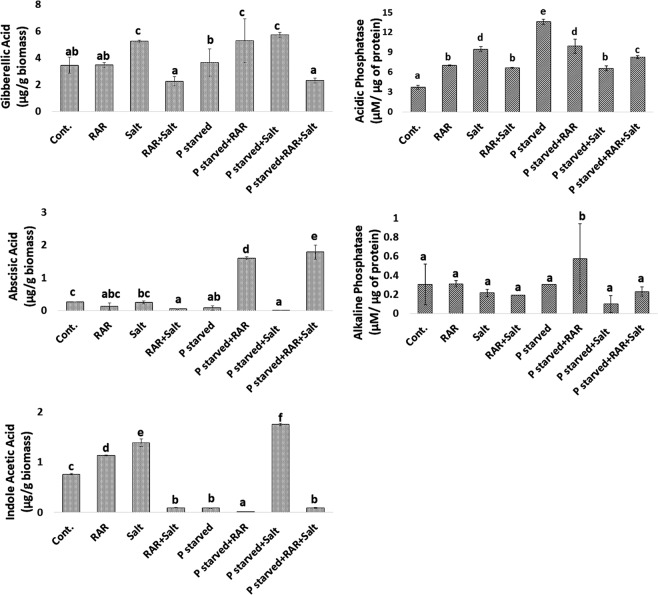
Effect of *Pseudomonas putida* MTCC 5279 (RAR) inoculation on endogenous level of hormones *viz*. gibberellic acid (**A**), indole acetic acid (**B**) and abscisic acid (**C**) in shoot and phosphatase activity; alkaline phosphatase (**D**) and acidic phosphatase (**E**) in roots of *A. thaliana* grown under salinity, phosphate starved and phosphate starved-salinity stressed (NaCl, 200 mM) condition. Vertical bars indicate mean ± S.D. of three replicates.

Increased gibberellic acid content was found under salinity and combined stress of both (P starved-salinity), however, P starvation alone didn’t modulate the GA content (Fig. 7A). Less accumulation of GA in presence of RAR under salt and P starved-salinity stress condition is probably associated with its own GA producing property as observed under *in vitro* condition. Higher concentration of ABA in presence of RAR under P starvation and P starved-salinity stressed condition has been observed (Fig. 7B). Higher IAA accumulation in plants in presence of RAR, salinity stress and P starved salinity stress condition was found. However, in presence of other stress conditions no such alteration was observed in presence of RAR (Fig. 7C).

### Effect of *P. putida* inoculation on phosphatase activities in *A. thaliana*

Salinity (200 mM NaCl) and P starved conditions raised the acidic phosphatase activity as compared to control conditions, however, alkaline phosphatase activity didn’t modulated under such conditions (Fig. 7D,E). Presence of phosphatase producing bacteria (RAR), was found to reduce both alkaline and acidic phosphatase activity under salinity stress (200 mM NaCl) and P starved alone conditions. Contrary to this both acidic and alkaline phosphatase activity was higher in presence of RAR under combined stress conditions (P starved-salinity).

### Effect of *P. putida* inoculation on gene expression in *A. thaliana*

**Figure 8:**
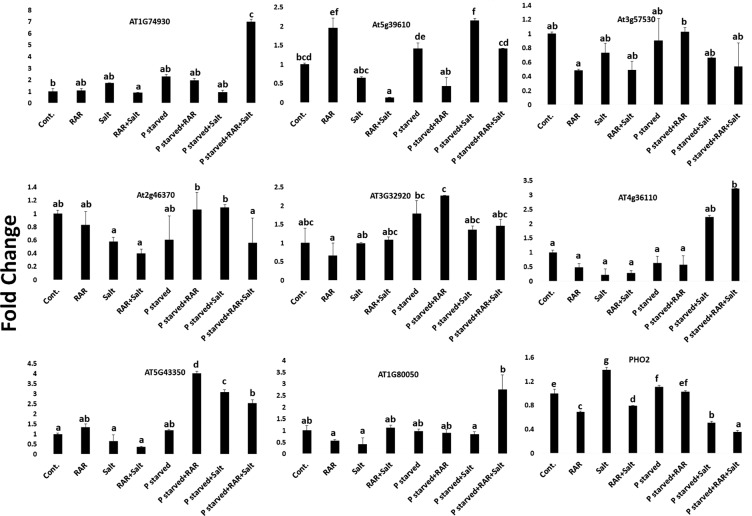
Effect of *Pseudomonas putida* inoculation on expression of randomly selected genes in *A. thaliana* root grown under salinity, phosphate starved and phosphate starved-salinity stressed (NaCl, 200 mM) condition. Vertical bars indicate mean ± S.D. of three replicates.

Real time PCR analysis of the randomly selected genes in the *A. thaliana* roots was studied under conditions of P starvation, salinity and P starved-salinity, both in presence and absence of RAR (Fig. 8). Expression of ethylene responsive transcription factor (*AT1G74930*; *AP2/ERF* family) gets modulated under P and salinity alone stress conditions, however, combined stress of both resulted in similar expression as compared to control. Presence of abiotic stress tolerant *P. putida* altered its expression under P starved (~2 fold) and combined stress conditions (~6 fold) as compared to control. *At5g39610*, encoding, *NAC*-domain transcription factor is known to positively regulate ageing-induced cell death and senescence in leaves. Present study showed overexpression under P starvation and combined stress conditions (P starved-Salinity), however, presence of *P. putida* was found to regulate its expression. *P. putida* alone has elevated its expression under control conditions probably associated with early maturing. Calcium-dependent protein kinase (*CPK32*, *At3g57530*), known to have role in signal transduction pathway in a Ca^+2^ dependent manner and regulate the expression of ABA responsive genes thereby helping in stress adaptation. Down regulation of *CPK32* under salinity and P starved-salinity conditions was observed which was further repressed in presence of RAR. Down regulation of jasmonate responsive gene (*JAR1, At2g46370*) in the root tissues under P starved and salinity stress alone condition has been found, whereas, presence of RAR modulates its expression similar to control only in P starved condition. Putative DNA repair protein, (*AT3g32920*), has been found overexpressed only in P starved and P starved + RAR conditions. Combined stress both in presence and absence of RAR found to elevate the expression of *At4g36110* by 2–3 fold. Expression profiling of different P transporters *viz*. *PT1 (AT5G43350), PT2 (AT1G80050)* and *PHO2* having role in P uptake under stressed conditions has also been performed. *PT1*, getting mildly over-expressed (1.5 fold) under normal P + RAR conditions, got upregulated by 3–4 fold under P starved conditions in presence of *P. putida*. Higher expression of *PT1* was found under combined stress condition in both presence and absence of RAR. However, higher expression of *PT2* was observed in combined stressed conditions only in presence of *P. putida*. RAR also modulated the reduced expression of *PT2* under salinity stress condition similar to control. *PHO2*, the gene responsible for P accumulation in shoot, got upregulated only under salt stress condition. While, under combined stress (P starved-Salinity), its expression got downregulated both in presence and absence of RAR.

## Discussion

Plant growth and productivity is continually decreasing due to occurrence of many biotic and abiotic stresses^30^. Development of methods for improved plant growth and productivity under stress condition play pivotal role for enhanced agricultural production. Deployment of rhizospheric microorganisms for improved growth and development is one of the ways to evoke the above problem^31^. The present study is based on the characterization of abiotic stress tolerant P-solubilising, auxin producing, and plant growth promoting *P. putida* MTCC 5279 (RAR)^29,32^, for production of proline, phosphatase enzyme and P accumulation (Fig. 1; Supplementary Fig. S2A–C and S4A,B). The work also elucidates the role of RAR for amelioration of salinity stress during P starved conditions through modulation of physiological processes correlated with the expression of different genes related to hormone synthesis, calcium dependent signalling and P transporters.

Unavailability of phosphorus often limits plant growth in a natural environment. Microorganisms are integral part of soil phosphorus (P) recycling, which is known to contribute plant P nutrition^8,19^. Modulation in acidic phosphatase activity in present study under both salinity and P starved alone conditions found to be ameliorated to some extent by the presence of RAR, supports the earlier report of Richardson *et al*.^33^. *P. putida* induced modulation of the acid phosphatases for maintenance of internal Pi concentration depicts its role for enhancing the P availability. Enhanced P availability is known to lower nutrient imbalances and energy crisis induced due to salinity stress in plants as reported earlier^8,10^. Earlier report also showed increased acid phosphatase activities for maintaining a certain level of phosphate in plant cells by catalysing non-specific hydrolysis of Pi from phosphate monoesters^34,35^.

Salt stress results in osmotic defects, ion toxicity, hormonal imbalance, generation of reactive oxygen species, membrane disorganization, inhibition of photosynthesis and attenuated nutrient acquisition in plants^6,36–38^. Reduced P accumulation under salinity (200 mM) and P starvation in present study supports the view of attenuated acquisition of essential nutrients under stress conditions. Salt induced reduction of P correlates well with the earlier report that phosphate availability in soil solution are tightly controlled by sorption process, availability of plant labile P and competition of Na^+^ and Cl^−^ with nutrients such as K^+^, Ca^2+^, H_2_PO_4_^−^ and NO_3_^−^^39–41^. Enhanced salt tolerance by P enrichment and treatment with phosphate solubilizing bacteria such as *Bacillus* and *Pseudomonas* spp. has been reported earlier^28,42,43^. The present study also showed the capability of RAR to improve growth of model plant *A. thaliana* under stressed conditions of salinity (200 mM), P starvation and combined stress of both. This observation may be attributed to the proline and auxin producing, P-solubilising and P accumulating ability of RAR. Role of auxin producing and P-solubilising bacterium for plant P uptake has been reported earlier^29,44^. Exogenous application of proline is also reported to alleviate salinity induced disturbances in plant^45^. Application of RAR in present study probably act as a source of proline for plants under stress condition.

The concentration of auxin and its ratio to other hormones are critical for the physiological responses of the plant^46^. Bacteria which produce IAA can add to, or influence the levels of endogenous plant auxin and promote growth under different stress conditions^47^. Auxin synthesis by rhizospheric bacteria often dependent on environmental tryptophan, stimulates root growth and branching^48^. Less auxin and gibberellin production by RAR under stress conditions, more pronounced in absence of TPP under *in vitro* conditions supports the earlier perspective that salinity stress restricts the production of IAA by reducing the production of precursors as evident from the correlation observed under IAA, and GA production with normal P under TPP deficient conditions^49^ (Fig. 1, Supplementary Fig. S4A,B). Similar pattern of gibberellic acid and auxin production under *in vitro* conditions, indicative of some common link between gibberellins and auxin production is in accordance to the earlier report that auxin and gibberellin (GA) often act synergistically by affecting the concentration of each other as a key signals involved in growth^50^ (Supplementary Fig. S4A,B). Increase in root production has also been correlated with better acquisition of phosphorus with number of physiological factors *viz*. lateral root branching and elongation, root hair density, organic acid exudation and phosphatases^33^. Better P content in presence of RAR in control, salinity stress and P starved saline stress conditions in present study supports the earlier report that presence of microbial inoculants influence the P uptake by root exudation and phosphatase activity^33,44^. Correlation among different morpho-physiological factors *viz*. root length, shoot length, dry weight, no. of siliques and P content with Salt + RAR conditions emphasizing more efficient role of RAR for amelioration of salinity stress as compared to combined stress conditions (Fig. 4).

*Arabidopsis* grown under limiting P conditions are known to reduce the primary root length, increased lateral root and root hair formation^51^. Cluster root formation in P depleted conditions and enhanced phosphatase activity due to cluster root formation has also been reported^52^. Present study also shows clustering of roots under P deprived conditions both in presence and absence of RAR, well correlated with higher phosphatase activity. However, under salinity alone and combined stress (P starved-salinity) conditions, lesser number of lateral roots accompanied with lesser root density is contrary to the report of Tang *et al*.^53^. ABA, as a potential chemical signals in auxin independent manner is known to modulate the root system architecture under water stress^54,55^. RAR mediated better root density observed in present study, also found to be associated with higher ABA content. Moreover, generation of ROS in response to abiotic stresses was also known to influence auxin response^56^. Present study also showed correlation between superoxide radical generation and IAA production under salinity and phosphate starved conditions. Reduced growth of *A. thaliana* under salinity stress condition was in accordance to the report of Ribaut and Pilet^57^, which showed reduced plant growth under increased IAA conditions. The variation in IAA content similar to ABA content has been reported under salinity condition^58^, however, contrary to earlier report, present study showed opposite pattern of IAA and ABA production under stress condition.

Plants exposed to abiotic stresses exhibit characteristic of rapid accumulation of GA for maintenance of physiology and metabolism to regulate the metabolic process as a function of sugar signaling and antioxidative enzymes^59^. Present study also reports the higher accumulation of GA3 under different stress conditions of salt and phosphate. Less accumulation of GA3 in presence of *P. putida* under salinity and P starved-salinity stress condition is probably associated with its stress ameliorative property. Induced acid and alkaline phosphatase activity under higher ABA, GA3 and NaCl stress has been reported in sorghum^60^. Higher GA3, ABA and phosphatase activity has also been investigated in the present study under salinity stressed conditions (Fig. 7A–D). Enhanced ABA in presence of RAR under P starvation and P starved-salinity stressed condition shows opposite pattern with H_2_O_2_ production as reported earlier, that higher H_2_O_2_ production is associated with higher ABA^61^.

Salinity stress is known to increase the level of ROS in the plant tissues due to irregularities in the electron transport chain and accumulation of photoreducing power^62^. Present study also reported higher accumulation of H_2_O_2_ and superoxide radicals in plants under stress conditions. Higher redox content under mineral deficient condition has also been reported earlier^51^. Present study also reports the elevated H_2_O_2_ in order to keep the redox status high for more nutrient availability (Fig. 6A). Higher redox state in presence of *P. putida* under drought has also been reported by Ghosh^63^. Elevated H_2_O_2_ in presence of RAR under control and salinity alone conditions supports the stress mitigation attribute of RAR to keep the nutrient availability high. Accumulation of proline under stress condition is a common phenomenon, which contributes to ROS scavenging, stabilization of subcellular structures, modulation of cell redox homeostasis^64^. Present study reports significant higher accumulation of proline under salt and P stress condition, further augmentation in presence of PGPR (RAR) shows the potential role of osmolytes in stress mitigation^64^. Antioxidative defense system of plants are known to subdue intracellular ROS by the action of defense enzymes. Among two different enzymes assessed in present study, catalase activity was profoundly higher under stress condition, whereas, no such modulation was observed in APX activity. Contrary to that, earlier study showed opposite trend of APX and catalase content in plant under salinity stress condition probably due to difference in sampling time^45^. Under salinity and drought stress conditions higher proline and antioxidative enzymes in presence of different PGPR in different crop plants has been reported^63,65^. Higher proline, catalase and APX activity in P starved and P starved-salinity stress condition in presence of RAR suggestive of the involvement of osmoprotectants and antioxidant enzymes for abiotic stress tolerance as per the earlier report^45,63,65^. Enhanced superoxide radical accumulation in presence of RAR under salt stress conditions is in accordance to the earlier report^63^, however, under P starved and combined stressed conditions, ameliorative property of RAR is evident by reduced NBT staining.

In present study modulation of randomly selected genes in *A. thaliana* grown under salinity and P deprived conditions in presence of *P. putida* has been studied (Fig. 8). Ethylene (ET) responsive transcription factors (ERF) are known to regulate different aspects of plant growth, development and their response to different stresses. Elevated expression of ERF under salinity stress (*AT1G74930*, an *AP2/ERF* family transcription factor) in present study, indicates ERF as a positive regulator of salt tolerance in *Arabidopsis* as per the earlier report^66^. Modulated expression of ERF, interpret that *P. putida* mediated repression under alone stress conditions suggestive of ET suppression resulting in better root architecture as evident by Camehl *et al*.^67^, though the capability of RAR was not enough to reduce the ethylene level under combined stress condition. Elevated expression of *CPK32 (At3g57530)* under stress conditions for increased cellular Ca^2+^ is crucial for plant defense against various stresses^68,69^. However, in present study downregulated expression of *CPK32* in root tissues both under stress and presence of PGPR was found to be different as reported earlier for leaf tissue^70^. Reduced expression of jasmonate responsive gene (*At2g46370*, *JAR1*) under salinity and P starved-salinity condition demonstrate RAR mediated ISR under salinity stress condition. However, decoy of jasmonic acid signalling pathway under combined stress conditions might work as suggested by Staswick^71^, that *JAR1* mutant showed reduced sensitivity to JA. *At4g36110* has been correlated to IAA production during plant-microbe interaction in *Arabidopsis*^29,72^. Upregulation under combined stress conditions in presence of RAR doesn’t correlates with better auxin production, on the contrary, ABA production was higher. Upregulation of *NAC* transcription factor under ABA and abiotic stresses, including high salinity, drought and low temperatures has already been reported^73^, present study also showed upregulation in P starved and P starved-salinity stress condition. However, correlation with ABA under combined stress condition, only in presence of RAR was observed. Repressed expression of *At3g32920* (putative DNA repair protein) in presence of RAR is as per the earlier report of Srivastava *et al*.^29^, however, its higher expression under P starved and P starved-salinity stressed conditions has been reported first time. RAR mediated overexpression under such conditions demonstrate its role in abiotic stress mitigation. Overexpression of P transporters in *A. thaliana* has been reported to grow better in P starved conditions by translocating Pi from the external media to the cytoplasm^74,75^. High affinity Pi transporters are inducible, whereas, the low-affinity transporter remains constitutive during Pi starvation^76^. Sodium dependent P uptake has been studied in *Zostera marina* by Rubio *et al*.^77^. *PT1* (*At5g43350*), the only phosphate transporter expressed in root is known to be overexpressed during P starvation^78^. Overexpression in P starved alone and combined stress in present study is well correlated with plant P uptake^78,79^. Low affinity H+/Pi chloroplastic cotransporter, *PT2* (*AT1g80050*), known to be involved in inorganic phosphate (orthophosphate, Pi) uptake in green parts of plants during P starvation^62^. Overexpression of this gene under combined stress conditions in presence of RAR, well correlated with higher P content of the shoot. *Pho2* involved in phosphate starvation response are known to play important role in Pi signalling during P starved condition. *Pho2* mutant are known to accumulate higher P content in shoot^80^. Present study also showed repressed expression correlated with higher P content of the shoot in presence of RAR under all conditions, thereby depicting its role for enhanced P uptake.

Study concludes that *P. putida* promotes the growth of *A. thaliana* under different stress *viz*. salinity, P starved and P starved-salinity stressed conditions through different biochemical and physiological modifications in plant. Different attributes of RAR such as proline and hormone production, phosphatase activity and P accumulation correlates well with modulated activities of plant. PGPR mediated modification in root architecture, ABA signalling, redox status, acid phosphatase activity and modulation of phosphate transporters probably associated with enhanced P uptake in plants for better survival under P starved-salinity stress conditions. However, RAR was found to modulate the salinity stress more efficiently as compared to the combined stress as evident from the correlation found among different morpho-physiological factor with salt + RAR conditions. Therefore, in order to feed the growing population of earth which is adding 83 million people every year at the rate of 1.09%^81^, present study provide the basis for the development of eco-friendly sustainable approach to increase agriculture productivity under stress conditions of salinity and P starved salinity.

## Materials and Methods

### Microbial growth conditions

*Pseudomonas putida* MTCC5279 (RAR), a P-solubilizing, auxin producing, abiotic stress tolerant bacteria^29^ grown in nutrient broth (NB) medium at 28 °C for 16 h was used as inoculum (@1%) for *in vitro* experiments. Salinity tolerance of the strain was performed by growing in M9 minimal salt medium supplemented with sodium chloride (NaCl; 0, 100, 200, 300, 400 and 500 mM). Cultures were incubated on a rotatory shaker at 28 °C, 180 rpm for 10 days followed by their sampling at 1, 3, 5 and 7^th^ day of inoculation. Log_10_ CFU/ml of the cultures grown under different conditions was determined by serial dilution method. For inoculation of *A. thaliana* (plant growth promotional assays), *P. putida* MTCC5279, RAR grown in nutrient broth at 28 °C for 48 h in a rotary shaker at 180 rpm, spun and resuspended in 20 mM magnesium sulphate (final density of 10^9^ CFU ml^−1^) was used as inoculum.

### Characterization of *Pseudomonas putida* MTCC5279

#### Phosphatase activity

Alkaline and acidic phosphatase activity of the Pseudomonas putida MTCC5279 (RAR) were determined by growing the bacterial culture in Erlenmeyer flask containing M9 media supplemented with different concentrations of phosphate [(1.2% Na_2_HPO_4_ + 0.3% KH_2_PO_4_ (normal P), 0.3% KH_2_PO_4_ (limited P) and 1.5% tri calcium phosphate (TCP, unavailable P)] and salt (500 mM and 1 M NaCl)]. RAR was inoculated (@1%) and flasks were incubated at 28 °C for 48 h on rotatory shaker (180 rpm). After determination of Log_10_ CFU/ml, phosphatase activity in the cell suspension was determined as per the protocol of Tommassen and Lugtenberg^82^. In brief, 1 ml bacterial culture was mixed with 250 µl of toluene, vortexed well and incubated at 28 °C for 2 h in shaking condition. Alkaline and acidic phosphatase activity was determined in a 3 ml reaction mixture containing, 500 µl of cell suspension (bacterial culture + toluene mixture); 200 µl of 0.1 M Tris pH 8.0 (for alkaline phopshatase)/Acetate buffer pH 5.2 (for acidic phosphatase) and 300 µl of 10 mg/ml para-nitrophenyl phosphate (pNPP; prepared in same buffer). Reaction mixture was incubated at 37 °C for 30 min and 1 ml of 3 N NaOH was added to stop the reaction, followed by absorbance at 420 nm.

#### Proline production

Proline accumulation property of the *Pseudomonas putida* MTCC5279 (RAR) was determined by growing the bacterial culture in Erlenmeyer flask containing M9 media with different phosphate sources [(1.2% Na_2_HPO_4_ + 0.3% KH_2_PO_4_ (P-normal), 0.3% KH_2_PO_4_ (limited P) and 1.5%Tri calcium phosphate (TCP, unavailable P)] in presence and absence of tryptophan and salt stress (NaCl, 500 mM and 1 M). Bacterial culture was inoculated (@1%) and cultures were incubated at 28 °C for 48 h on rotatory shaker (180 rpm). Proline accumulation was estimated in culture supernatant as per the method of Bates *et al*.^83^. In brief, one ml of culture supernatant was mixed with equal volume of glacial acetic acid and ninhydrin reagent (1.25 g ninhydrin dissolved in 20 ml glacial acetic acid and 6 M of 30 ml orthophosphoric acid), incubated for 45 min in a boiling water bath followed by snap chilling in ice to stop the reaction. Reaction mixture was added with 2 ml of toluene, vortexed well and absorbance of the upper toluene layer was recorded at 520 nm using UV-Vis spectrophotometer. To elucidate the role of proline as an ATP dependant phenomenon, effect of ATP inhibitor carbonyl cyanide-m-chlorophenylhydrazone (CCCP) was studied under salt stress condition as described earlier^84^. In brief 1.6% agar supplemented plates of M9 minimal media containing 0, 500 and 1000 mM NaCl in presence of proline (10 µM) and CCCP (50 µM) were prepared and RAR was streaked. Plates were incubated at 28 °C for 48 h. Development of intense pink color is indicator of proline production.

#### Determination of accumulated Phosphate

Effect of different phosphate sources on phosphate accumulation property of RAR was determined by growing the bacterial culture in NBRI-PA media with varying P sources (1.2% Na_2_HPO_4_ + 0.3% KH_2_PO_4_, 1.5% hydroxyapatite and 1.5% TCP). Bacteria was inoculated (@1%) and flasks were incubated at 28 °C for 48 h. Bacterial cells were harvested through centrifugation (10,000 g for 10 min) and accumulated Pi in the bacterial biomass was extracted as described earlier by Chaudhry and Nautiyal^85^. Extracted P was estimated by molybdenum blue method^86^. In brief, 100 µl of extract was mixed with equal volume of 2 N HCl followed by 30 min incubation in a boiling water bath. After incubation, 700 µl of molybdenum blue solution (30 ml of 0.42% ammonium molybdate in 1 N H_2_SO_4_ mixed with 500 mg of sodium ascorbate prepared in 5 ml MQ) was added and reaction mixture was incubated at 45 °C for 20 min after dilution to 5 ml with sterile MQ. Final absorbance was taken at 820 nm using UV-Vis spectrophotometer. Standard was prepared using KH_2_PO_4_ and accumulated Pi was calculated in terms of biomass. Correlation between accumulated Pi and proline was determined in PAM media in presence of ATP inhibitor CCCP (50 µM) under salt (500 mM and 1 M) stress condition as described above.

#### Hormone analysis in bacterial culture

Hormone analysis was performed in bacterial culture grown for 48 h in M9 media supplemented with different concentration of salt (0, 500 mM and 1 M salt) in presence and absence of tryptophan with variable phosphate sources (1.2% Na_2_HPO_4_ + 0.3% KH_2_PO_4_ (P normal), 0.3% KH_2_PO_4_ and 1.5% TCP). Hormone was extracted from cell free culture supernatant after adjusting the pH of supernatant to 2.8 using 1 N HCL followed by its liq/liq extraction with ethyl acetate (step was repeated for three times). Both inorganic (aqueous) and organic (ethyl acetate) phase were separated using separating funnel^87^. Organic phase was dried under vacuum and IAA and GA was analysed using HPLC as described earlier by Srivastava *et al*.^88^.

### Effect of *P. putida* inoculation on amelioration of phosphate and salinity stress on *A. thaliana*

Plant growth promotary effect of *Pseudomonas putida* MTCC5279^29^ under salt, P-starvation and combined stress of salinity and P was assessed under both *in vitro* and *in vivo* conditions.

### *in vitro* plant growth assays

To observe the effect of RAR inoculation on morphological alterations of *A. thaliana* grown under different growth conditions of salinity, P- starvation and combined stress of both, *in vitro* experiments were performed. The plants were grown in MS^89^ (0.5×) media, supplemented with 1% sucrose, final pH adjusted to 5.6 followed by addition of 0.8% agar wherever required. Surface sterilized pre-germinated one week old seedlings of *A. thaliana* was transferred in 0.6 ml eppendorf tubes dipped in 1.5 ml tube containing MS (Supplementary Fig. S6D) or plants were grown in petriplates on nets placed on sterile foams saturated with MS media as per different treatments (Supplementary Fig. S6F) (hydroponic condition). Treatment of RAR (@1% v/v, O.D.−0.6) to the *A. thaliana* grown under different stress conditions in MS media under hydroponic condition was given after 3^rd^ day of seedling transfer. Phosphate starvation was given after 7 days of bacterial inoculation by transferring the plants into fresh media deprived of any P source and after 3 days of P starvation, salt stress (NaCl; 200 mM) was given and photographs were taken after 5 days of salt stress. Alternatively plants were also grown in ½ MS media plates carrying different salt concentration in order to see the effect of salinity on germination of *A. thaliana* seeds (Supplementary Fig. S5A). Inoculation of *P. putida* MTCC 5279 on ½ MS media plates was performed by filling the wells lacerated in agar plates on which one week old seedlings were transferred in a single line (Supplementary Fig. S6A,C).

### *In vivo* plant growth assays

To study the physiological alteration in *A. thaliana* grown under different growth conditions of salinity, P starvation and combined stress of both P and salinity, *in vivo* experiments was performed under pot conditions. Surface sterilized seeds of *A. thaliana* was sown in sterile soil-rite mixture, followed by transfer in growth chamber after stratification (72 h at 4 °C). RAR treatment was given by inoculation of log phase grown culture of RAR (spun and resuspended in 20 mM magnesium sulphate, O.D. ~0.6 corresponding to ~10^9^ cfu/ml), around the plant roots after fifteen days of germination (at four leaf stage). The control set were inoculated with same amount of sterile 20 mM magnesium sulphate. Plants were irrigated weekly with the nutrient solution^90^ (OS media). Phosphate stress was given to the plants after 15 days of bacterial inoculation by replacing KPO_4_ buffer with K_2_SO_4_ in OS media. After 15 days of P stress, salt treatment were given twice in a week by irrigating with nutrient solution containing salt (NaCl; 200 mM) and were harvested after 5^th^ day of second salt treatment. Treatments were (i), control; (ii), P starved; (iii), RAR; (iv), P starved + RAR; (v), salinity (NaCl, 200 mM); (vi), salinity + P starved; (vii), salinity + RAR and (viii), RAR + salinity + P. All experiments were conducted in a temperature controlled growth chamber (16/8 light/dark conditions, 22 °C temperature and 70% relative humidity). Plant growth promotional effect of *P. putida* on *A. thaliana* grown under control and stress condition was evaluated by measuring the root length, shoot length, number of branches, number of siliques and dry weight of the 4 plants from each replicates and each treatment has 6 replicates. Data provided is a mean±s.d. of 24 plants.

### Effect of *P. putida* on physiological alteration in *A. thaliana*

Different physiological parameters in *A. thaliana* grown in soil rite under conditions of salinity, P starvation and combined stress of both was assessed to evaluate the stress ameliorative effect of RAR.

### Proline, phosphate (P) and chlorophyll content

Proline accumulation in shoot of *A. thaliana* plant grown under P and salt stress condition in presence of RAR under soil rite pot conditions was estimated as per the protocol of Bates *et al*.^83^. Plant tissue was macerated in 3% sulfosalicylic acid and the supernatant obtained after centrifugation was mixed with the equal volume of acid ninhydrin reagent and glacial acetic acid as described above.

P acquisition in shoot tissue of *A. thaliana* grown in soil rite was assessed by ammonium molybdate blue method. Plant tissue (0.1 g) was homogenized in 0.5 ml of 10% perchloric acid. Supernatant obtained after centrifugation (10,000 rpm for 10 min.) was mixed with acetate buffer (0.1 M, pH 5.0) followed by ammonium molybdate (1%, prepared in 0.5 N H_2_SO_4_) and sodium ascorbate (1%) as described earlier by Tsvetkova and Georgiev^91^. Final absorbance was taken at 620 nm. Photosynthetic pigment was estimated in leaf as described earlier by Srivastava *et al*.^88^. In brief, 0.05 g of shoot was crushed in 80% chilled acetone followed by centrifugation at 10,000 rpm for 10 minutes. Absorbance was taken at 663 and 645 nm.

### Root phosphatase activity

Alkaline and acidic phosphatase enzyme activities of *A. thaliana* grown in soil rite was performed by crushing the roots (0.1 g) in 2.0 ml of 0.1 M phosphate buffer (pH 6.6). Activities were performed in a reaction mixture of 3 ml as discussed above for bacterial cells with their respective buffers (Acetate buffer pH 5.2 for acidic and Tris HCl buffer pH 8.0 for alkaline phosphatase) as per the protocol of Tommassen and Lugtenberg^82^.

### Defense enzyme activities

Activities of defense enzymes (catalase and APX) was determined by homogenizing 0.5 g of shoot tissue in 5 ml of extraction buffer [0.1 M, pH 7 potassium phosphate buffer containing, 0.1 mM EDTA, 1% polyvinyl pyrrolidone (PVP), PMSF (100 µM) and dithiothreitol (0.3%)]. The enzyme extract centrifuged and the activities of ascorbate peroxidase (APX) and catalase was determined in supernatant using the method of Nakano and Asada^92^ and Aebi^93^. In brief, APX activity was estimated by recording decrease in absorbance at 30 sec time interval upto 3 min at 290 nm in a 3 ml reaction mixture containing 0.1 M phosphate buffer (pH 7.0), 0.5 mM sodium ascorbate, 0.1 mM EDTA and enzyme extract. Catalase activity was measured by recording the decrease in absorbance of H_2_O_2_ at 240 nm in a 3 ml reaction mixture containing 50 mM of phosphate buffer (pH 7.0), 20 mM of H_2_O_2_ and enzyme extract.

### Histochemical detection of superoxide anion and hydrogen peroxide content

#### Superoxide anion (O_2_^−^)

Fresh leaves of *A. thaliana* grown in soil rite under different stressed conditions were transferred in nitroblue tetrazolium (NBT; 1 mg/ml) solution prepared in 25 mM HEPES; pH 7.6. Vacuum infiltration of the leaf immersed in NBT solution was performed for 5 min followed by incubation under dark conditions. After 2 h of incubation, leaves were decolorized using bleaching solution (ethanol:acetic acid:glycerol in 3:1:1 ratio) at 90–95 °C for 15 min^94^.

#### H_2_O_2_ detection

Hydrogen peroxide detection in leaves was performed through diaminobenzidine (DAB) staining. Fresh leaves of *A. thaliana* were immediately transferred in DAB solution, prepared according to Kumar *et al*.^95^. Vacuum infiltration of the leaf immersed in DAB solution was performed for 5 min followed by incubation for 4 h under dark conditions. After incubation leaves were decolorized using bleaching solution (ethanol:acetic acid:glycerol in 3:1:1 ratio) at 90–95 °C for 15 min^94^.

#### Hormone analysis in *A. thaliana* plants

Hormone extraction from the leaf tissue of *A. thaliana* grown in soil rite under different treatments *viz*. control, RAR, P starved, P starved + RAR, salt, salt + RAR, P starved + salt, P starved + salt + RAR was performed as described earlier^88^. In brief, group of hormones (gibberellic acid, indole acetic acid, and abscisic acid) were extracted in 3 ml of extraction buffer containing 2-propanol: H_2_O: Conc. HCl (2:1:0.002) using 300 mg of frozen tissues. The supernatant after centrifugation was carefully transferred to pre-cooled tubes, followed by addition of dichloromethane (1:2 ratio) and incubated for 30 min on rotary shaker at 4 °C. Lower phase of the samples was collected after centrifugation and concentrated through lyophilisation. Qualitative and quantitative analysis for separation of each of the methanol suspended extracts was achieved by HPLC-PDA with a LC-10 system comprising LC-10AT dual pump system, SPD-M20A PDA detector, and rheodyne injection valve furnished with a 20 µL sample loop (Shimadzu, Japan) as described earlier^32,88^.

#### Expression analysis of stress responsive genes

Total RNA of the *A. thaliana* root was extracted using RNA easy mini kit (Qiagen) according to the manufacturer’s instructions. To remove residual DNA, RNA was treated with DNaseI. Reverse transcription was carried out using 1 µg of the total RNA with revertaid H minus cDNA synthesis kit (Thermo) as per the manufacturer’s protocol. Real-time PCR analysis of randomly selected genes using actin as an internal reference was carried out in a 10 µL reaction mixture with Quanti-Tect TM SYBR® Green PCR kit (Qiagen) on Stratagene Mx3000P systems. The reactions were performed using the cycle conditions of an initial denaturation at 94 °C for 5 min, followed by 35 cycles of 94 °C for 30 s, 60 °C for 30 s, and 72 °C for 30 s. After obtaining ct value for each reaction, the fold change was calculated by delta-delta ct method (Table S1).

### Statistical analysis

One-way analysis of variance (ANOVA) and Duncan’s multiple range test (DMRT) were performed to identify the significantly different treatments using SPSS 16.0. All biochemical assays were performed in 4 replicates and data is given as mean ± s.d of triplicates. Biplot analysis (principal component analysis; PCA) was done using XLstat statistical software (version 2019.4.1).

## Supplementary information

Supplementary information.
