# Supplementary material for: Prescience of endogenous regulation in Arabidopsis thaliana by Pseudomonas putida MTCC 5279 under phosphate starved salinity stress condition
Source: Sci Rep. 2020 Apr 3;10:5855. doi: 10.1038/s41598-020-62725-1 (PMC7125087; doi:10.1038/s41598-020-62725-1)
Supplement: Supplementary file 1 — Supplementary information. [file 41598_2020_62725_MOESM1_ESM.docx]

**Prescience of endogenous regulation in *Arabidopsis thaliana* by *Pseudomonas putida* MTCC 5279 under phosphate starved salinity stress condition**

**Sonal Srivastava^1,2^, Suchi Srivastava^1,2^***

^1^Division of Microbial Technology, CSIR-National Botanical Research Institute, Rana Pratap Marg, Lucknow 226 001, India

^2^Academy of Scientific and Innovative Research, AcSIR, Ghaziabad- 201002, India

***For correspondence**

**Dr. Suchi Srivastava**

**e-mail** [**ssnbri@gmail.com**](mailto:ssnbri@gmail.com)

**Division of Microbial Technology**

**CSIR-National Botanical Research Institute**

**Rana Pratap Marg, Lucknow - 226 001, India**

**Phone: +91-522-2297988**

**Fax: +91-522-2205839**

Supplementary Table 1. List of primers used in the study.

| S/No. | Primer Details | Primer Sequence | Source |
| --- | --- | --- | --- |
| 1 | AT1G74930 | For: TTACGACACTCCCGAGAAGG | Srivastava et al., 2012 |
|  |  | Rev: TCCTCCGTAATCTTCGATGG |  |
| 2 | At5g39610 | For: AACCCTTCCCTCTTCGTCTC | Srivastava et al., 2012 |
|  |  | Rev: TCCAATAACCGGCTTCTGTC |  |
| 3 | CPK32  (At3g57530) | For: GTCGTGGAGAATTCGGTGTT | Srivastava et al., 2012 |
|  |  | Rev: GTTTCAGGTCCCTGTGCATT |  |
| 4 | JAR1  (At2g46370) | For: ATTGCAACTGTTTCGCACTG | Srivastava et al., 2012 |
|  |  | Rev: CGTGAACTAGACCGTGAGCA |  |
| 5 | AT3g32920 | For: ATCGGGAAAGACAGCACTTG | Srivastava et al., 2012 |
|  |  | Rev: CTGTTGGACCTCCAAATCGT |  |
| 6 | At4g36110 | For: TCAACACCGAAGTCGCTATG | Srivastava et al., 2012 |
|  |  | Rev: CCATTTGCTTCCGTCTTCTT |  |
| 7 | PT1  (AT5G43350) | For: TCTCAACGCCTCCTCAAGTT | This study |
|  |  | Rev: TTTGATGTTCTTGGCAACCA |  |
| 8 | PT2  (AT1G80050) | For: AAGGGAGAGTGATAAGCGAAGA | This study |
|  |  | Rev: CCCACCAGTGGCAACTAAAT |  |
| 9 | PHO2 | For: GCCATTCCCCCAAATTTACT | This study |
|  |  | Rev: GGATTCTGCAAAGCTTGAGG |  |

Fig. S1 Salt tolerance of *P. putida* MTCC 5279 grown under different concentration of salt (100-500 mM)


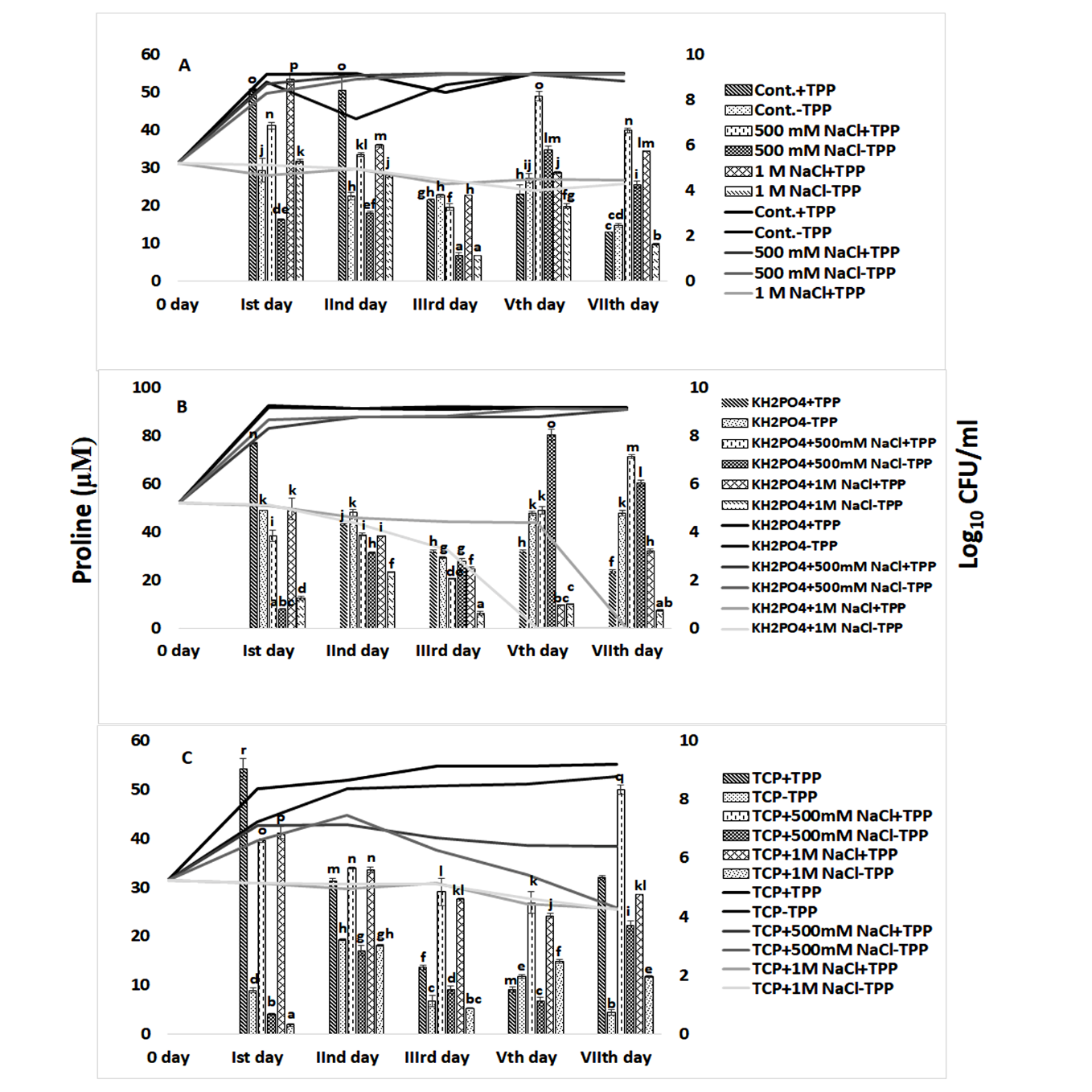


Fig. S2 Effect of salinity (0, 500 mM and 1 M NaCl) and different phosphate sources, (A) Normal P (0.3% KH_2_PO_4_+1.2%Na_2_HPO_4_), (B) limited P (0.3% KH_2_PO_4_) and (C) unavailable P (Tri calcium phosphate) stress on growth and proline accumulation property of *Pseudomonas putida* MTCC 5279 (RAR) in presence of auxin precursor tryptophan. Results are the means of three independent experiments. SD ± 0.25 log10 CFU/ml was found for the viable cell counts. Vertical bars indicate mean ± S.D. of three replicates.


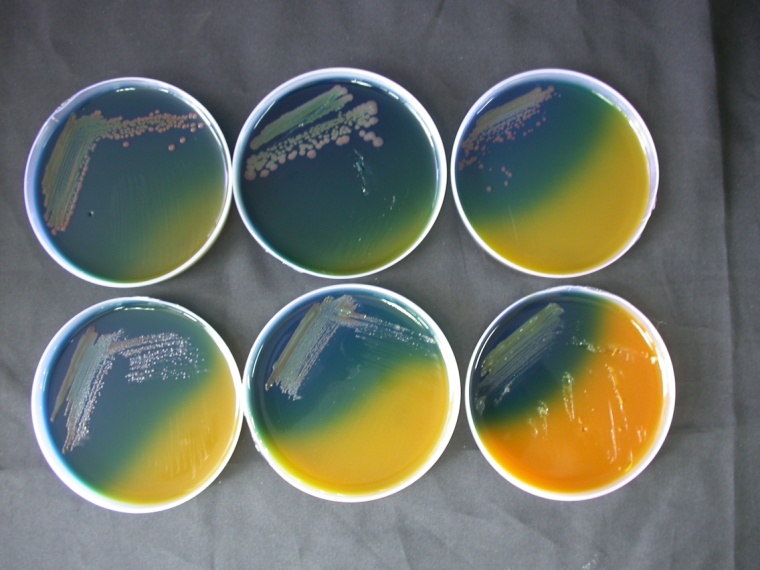


**C**

**-CCCP**

**+CCCP**

**RA RA+500mM NaCl RA+1 M NaCl**

Fig. S3. Accumulated Pi and proline in presence and absence of ATP inhibitor CCCP and salt (A) Qualitative (C)


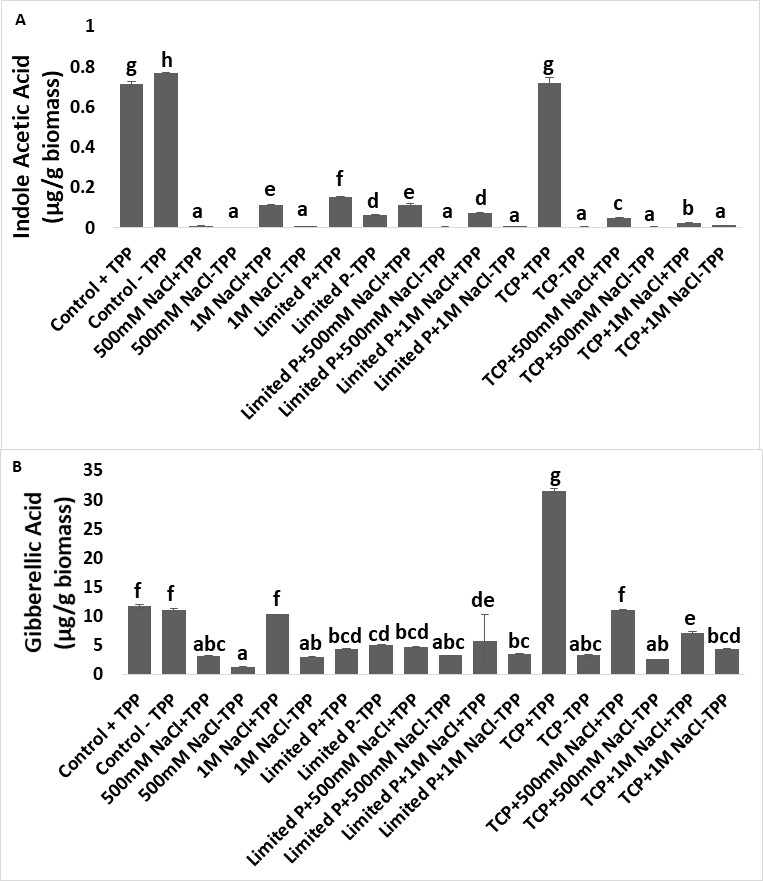


Fig. S4. Effect of salinity (0, 500 mM and 1 M, NaCl) and different phosphate sources [(Normal P (0.3% KH_2_PO_4_+1.2%Na_2_HPO_4_), limited P (0.3% KH_2_PO_4_) and unavailable P (Tri calcium phosphate)] stress on phosphatase activity growth hormones (C) indole acetic acid and (D) gibberellic acid of *Pseudomonas* *putida*. Results are the means of three independent experiments. Vertical bars indicate mean ± S.D. of three replicates.


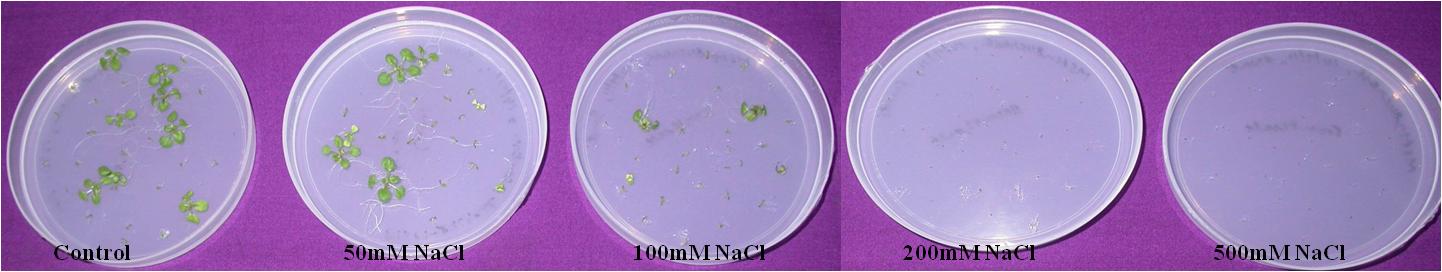


A

B

Fig. S5. Effect of salt (NaCl) on germination of *Arabidopsis thaliana* (A) and phosphate starvation alone and in combination of salt (200mM NaCl) on growth and hormone production of *Arabidopsis thaliana* (B)


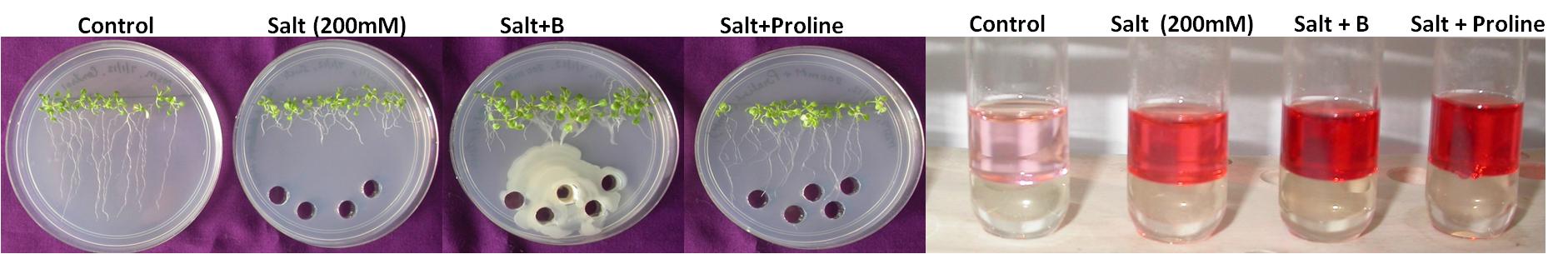


Fig. S6A. Effect of *Pseudomonas putida* inoculation on growth of *A. thaliana* plant as compared to the proline supplementation.

A

C

D


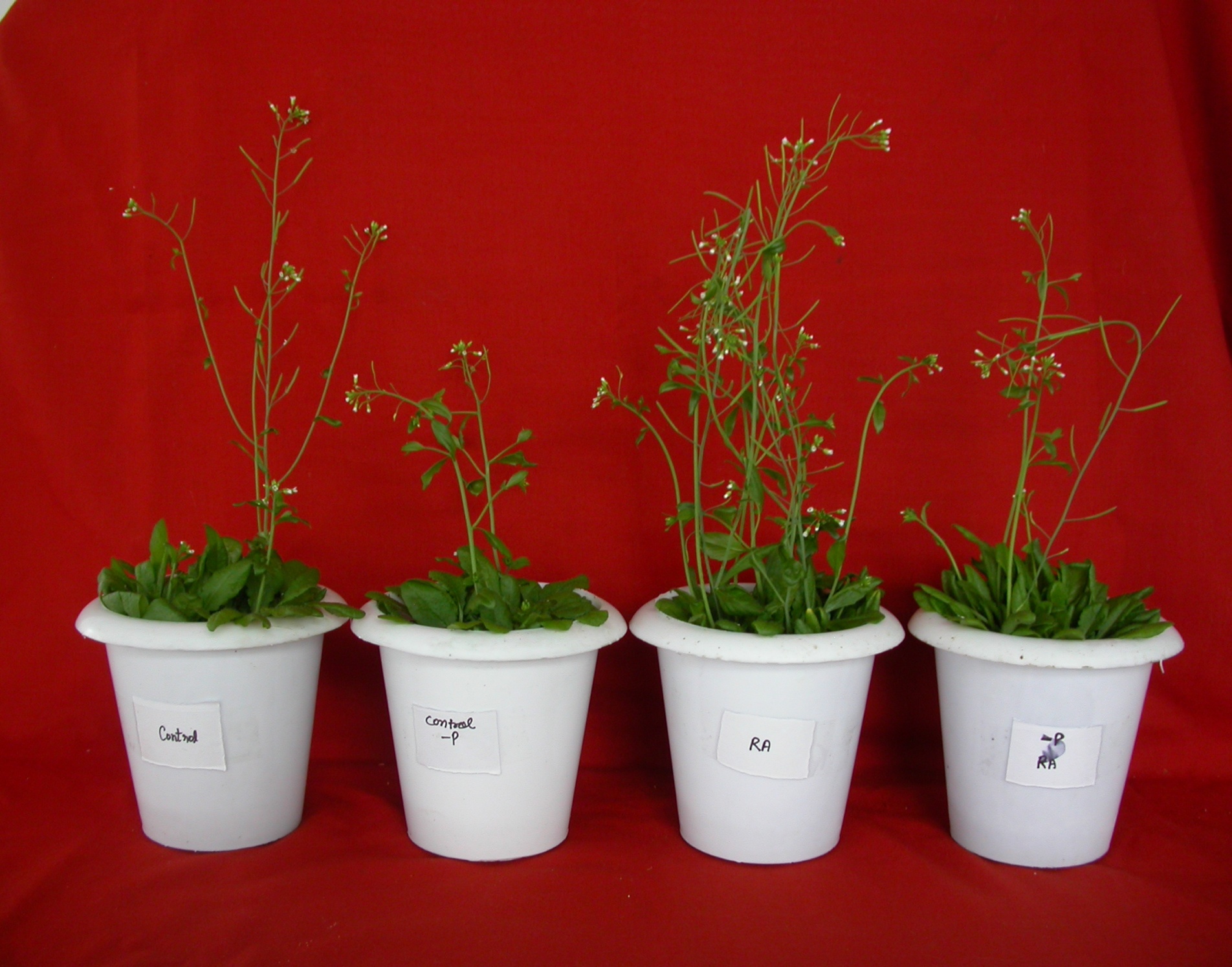

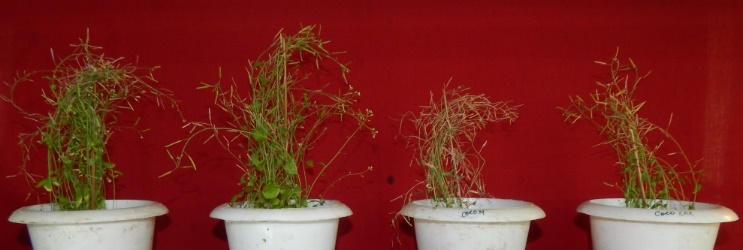


E

**CONT RAR P starved P starved+RAR**

F


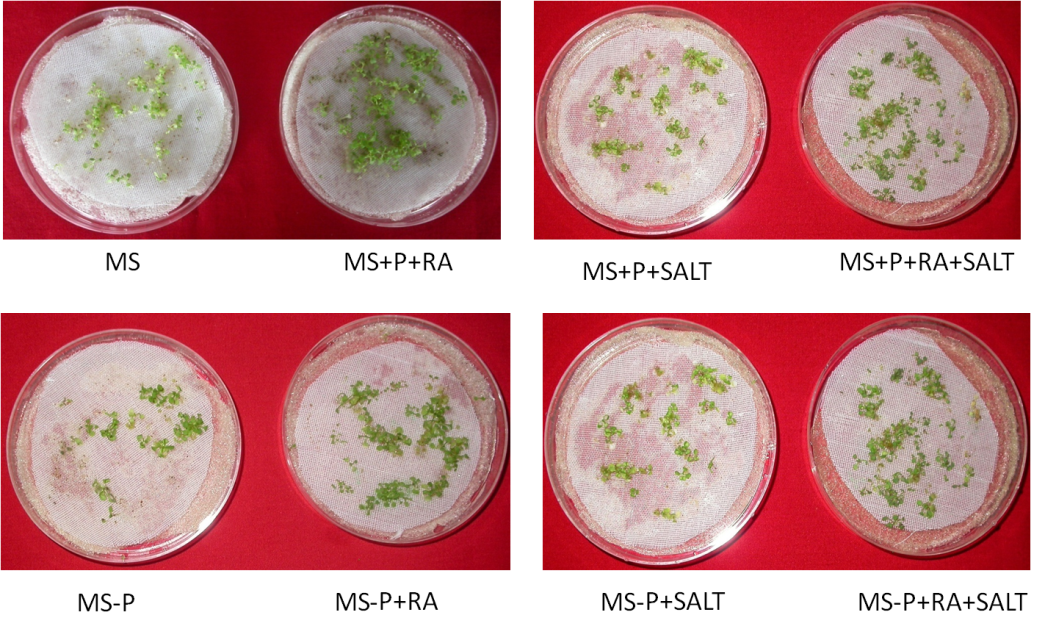

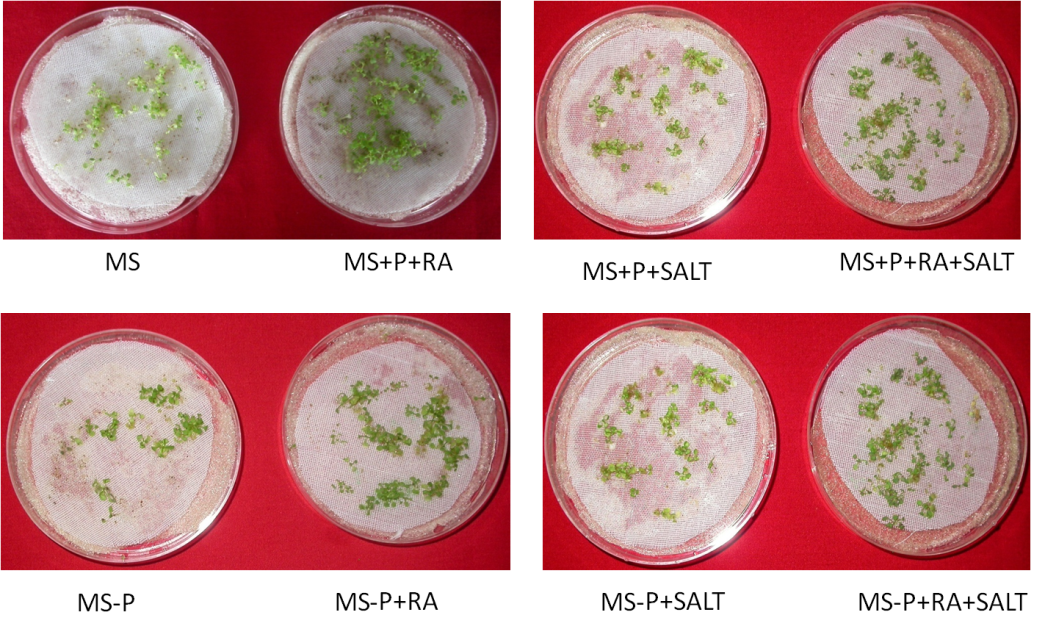


**CONT Salt (200mM) RAR Salt +RAR**

Fig. S6. Effect of *P. putida* RAR inoculation on *A. thaliana* grown under different growth conditions in phosphate starved and salinity stress condition

Supplementary Table 2. Effect of *P. putida* MTCC 5279 (RAR) inoculation on physical and physiological parameter of *A. thaliana* grown under phosphate starved and salt stressed (NaCl, 200mM) condition. ± indicate mean S.D. of three replicates.

|  | Control | RAR | Phosphate Starvation | Phosphate Starvation + RAR | Salt | Salt+RAR | Phosphate Starvation+ salt | Phosphate Starvation+ salt +RAR |
| --- | --- | --- | --- | --- | --- | --- | --- | --- |
| Shoot length (cm) | 21.25±4.27^b^ | 26.75±3.59^c^ | 22±0.81^b^ | 23.25±6.70^ab^ | 21.5±1.29^b^ | 23.5±1.29^ab^ | 15±5.59^a^ | 22±1.41^b^ |
| Root length (cm) | 8±0.70^b^ | 9.87±0.89^c^ | 9.75±2.04^c^ | 10±0.70^c^ | 7.75±0.43^b^ | 7.75±0.43^b^ | 6±0.70^a^ | 8.5±1.80^bc^ |
| No. of Leaves | 11.75±1.70^ab^ | 20.25±5.43^c^ | 12.75±2.75^ab^ | 13.5±1.73^b^ | 9.5±1.0^a^ | 14±1.41^b^ | 13.5±5.06^b^ | 12.5±2.08^ab^ |
| No. of siliques | 85±12.67^de^ | 103±14.76^e^ | 43.25±16.27^b^ | 65.75±32.80^cd^ | 54.5±16.25^bc^ | 105±16.83^c^ | 17±9.62^a^ | 62±19.30^bc^ |
| Dry Weight (g) | 0.43±0.032^b^ | 0.88±0.011^f^ | 0.26±0.011^d^ | 0.54±0.03^e^ | 0.24±0.00^b^ | 0.36±0.00^c^ | 0.19±0.00^a^ | 0.26±0.06^b^ |
| Chlorophyll A  (mg/g biomass) | 2.53±0.01^a^ | 2.49±0.02^a^ | 2.50±0.00^a^ | 2.49 ±0.08^a^ | 2.50±0.05^a^ | 2.49±0.01^a^ | 2.47±0.01^a^ | 2.48±0.01^a^ |
| Chlorophyll B  (mg/g FW) | 4.37±0.01^a^ | 4.55±0.02^cd^ | 4.67±0.01^c^ | 4.48±0.03^b^ | 4.52±0.03^c^ | 4.60±0.00^d^ | 4.53±0.04^a^ | 4.59±0.01^d^ |
| Total Chlorophyll (mg/g FW) | 6.89±0.03^a^ | 7.02±0.00^bc^ | 7.15±0.01^d^ | 6.95±0.12^ab^ | 7.02±0.08^bc^ | 7.07±0.01^cd^ | 6.98±0.02^abc^ | 7.06±0.02^bcd^ |
| Proline (µM) | 29.70±11.50^a^ | 43.47±15.58^a^ | 209.68±26.01^d^ | 111.95±11.71^bc^ | 56.07±7.41^ab^ | 107.41±38.52^bc^ | 133.51±0.80^c^ | 293.70±8.71^c^ |
| P-content (µg/g biomass) | 181.48±5.72^d^ | 198.12±5.72^c^ | 82.16±2.08^b^ | 199.68±3.12^e^ | 143±0.52^c^ | 226.72±5.2^f^ | 60.32±5.2^a^ | 183.56±6.76^d^ |

Fig. S7. Effect of *P. putida* inoculation on Catalase and APX activity in *A. thaliana* grown under phosphate starved and salt stressed (NaCl, 200mM) condition
